# Pain Acceptance and Its Associated Factors among Cancer Patients in Mainland China: A Cross-Sectional Study

**DOI:** 10.1155/2019/9458683

**Published:** 2019-02-13

**Authors:** Xianghua Xu, Meijun Ou, Chanjuan Xie, Qinqin Cheng, Yongyi Chen

**Affiliations:** ^1^Head & Neck Plastic Surgery, The Affiliated Cancer Hospital of Xiangya School of Medicine, Central South University/Hunan Cancer Hospital, Changsha, Hunan, China; ^2^Nursing Department, The Affiliated Cancer Hospital of Xiangya School of Medicine, Central South University/Hunan Cancer Hospital, Changsha, Hunan, China; ^3^Pain Ward, The Affiliated Cancer Hospital of Xiangya School of Medicine, Central South University/Hunan Cancer Hospital, Changsha, Hunan, China

## Abstract

**Background:**

Pain acceptance is associated with disability, pain interference, depression, and anxiety. Few studies have been conducted on the acceptance of cancer pain and its correlates.

**Objectives:**

The aim of this study was to examine the level and correlates of pain acceptance in cancer patients from mainland China.

**Setting and Participants:**

The study comprised 156 cancer patients in a tertiary cancer hospital in Hunan Province of China.

**Design:**

The study is based on a cross-sectional survey design.

**Subjects and Methods:**

The 8-item Chronic Pain Acceptance Questionnaire (CPAQ-8) was completed by 156 cancer patients with chronic pain from a tertiary cancer hospital. Demographics, pain, and negative mood assessed by the Hospital Anxiety and Depression Scale (HADS) were explored in relation to the CPAQ-8 scores using descriptive univariate analysis.

**Results:**

For the 156 patients, the mean CPAQ-8 score was 25.99 (SD = 8.56; range: 9 to 44). The scores were associated with age, gender, marital status, pain duration, number of pain sites, and duration of taking analgesics. The total scores on the CPAQ-8 and its two subscales (activity engagement and pain willingness) were negatively correlated with the HADS scores.

**Conclusions:**

The findings suggest that the prevalence of pain acceptance is relatively low for Chinese cancer patients. The cancer pain acceptance is affected by age, gender, pain duration, number of pain sites, and duration of taking analgesics. The acceptance of cancer pain is negatively correlated with depression and anxiety. Therefore, patients with risk factors for low pain acceptance should receive more attention in Chinese medical settings.

## 1. Introduction

In 1995, the American Pain Society (APS) voiced the slogan “pain: the fifth vital sign” to elevate awareness of pain treatment among healthcare professionals [1]. As the third global public health problem, chronic pain (CP) refers to pain or discomfort that has persisted continually or intermittently for longer than three months [2]. The prevalence of moderate to severe general CP among Dutch adults was estimated to be 18% [3], whereas estimates for Canada ranged from 11% to 44% of the adult population [4]. In a systematic review of 52 studies, the prevalence of cancer-related pain ranged from 33% to 64% of cancer patients, making it one of the most common and troublesome symptoms affecting patients with cancer [5]. Chronic pain is significantly associated with lower quality of life and higher psychological distress [6]. Moreover, pain could cause the onset of depressive or anxiety disorder in 15.5% of participants with no previous history of the disorder and no current depression or anxiety [7], and the coexistence of depression and anxiety with chronic pain is strongly related to more severe pain [8].

Cognitive behavioral therapy (CBT) is one of the best-known nonpharmacological interventions that have been studied extensively for chronic pain. An integrative review concluded that CBT reduced pain intensity in 43% of trials [9]. However, a review that applied strict inclusion criteria in identifying studies for analysis showed that CBT produced small effects on pain, particularly at follow-up, which may be caused by the lack of a clear treatment process within CBT [10]. In recent years, there has been growing interest in acceptance and commitment therapy (ACT), the third wave of CBT, which focuses on helping people disengage from unsuccessful efforts to control or avoid an unpleasant experience and instead accept it and move forward, pursuing valuable goals [11]. It has roots in learning theory and basic processes of language and cognition guided by relational frame theory [12]. ACT is based on the psychological flexibility model. Psychological flexibility has been defined as the capacity to persist in or change behavior, guided by one's goals that incorporate conscious and open contact with thoughts, feelings, and sensory experiences [12, 13]. The ultimate goal of ACT is to increase psychological flexibility through six core processes: acceptance, defusion, self-as-context, present moment, values, and committed action [11]. An ACT-based treatment for chronic pain patients could reduce levels of depression, pain-related anxiety, physical and psychosocial disability, and pain intensity and significantly increase psychological flexibility [14, 15]. In ACT-oriented interventions, psychological flexibility has been found to play a mediating role in improving functioning and life satisfaction in people with chronic pain [16].

Acceptance is a key part of ACT core processes. It involves the active and aware embracing of those private events occasioned by one's history without unnecessary attempts to change their frequency or form, especially when doing so would cause psychological harm [11]. An ACT cross-sectional study on participants with chronic pain found that acceptance may have a mediating effect on change in physical functioning [17]. Psychological flexibility is not easy to measure, but acceptance, which is related to psychological flexibility [18], can be assessed by a questionnaire. The higher a person's pain acceptance level, the better his/her psychological flexibility.

Pain acceptance is individuals' willingness to continue to actively experience pain along with related thoughts, feelings, and actions to move forward with their goals or act on their values while experiencing pain [19]. It can be assessed by the Chronic Pain Acceptance Questionnaire (CPAQ) [20, 21], which has been found to have the best overall reliability and validity among a number of pain acceptance questionnaires [22], including two subscales, pain willingness, and activity engagement. Pain willingness reflects efforts directed at controlling pain, while activity engagement reflects the degree to which a person continues to engage in personally meaningful activities despite pain.

A number of intervention studies have indicated that ACT can increase pain acceptance, improve quality of life, and decrease pain intensity and anxiety symptoms [23, 24]. Pain acceptance predicts depressive symptoms, pain-related negative affect, pain interference, performance in everyday living activities, inpatient hospitalizations, and painkiller consumption [25, 26]. Additionally, pain acceptance mediates the relationships between perceived injustice and physical function, opioid use status, and pain intensity [27]. Higher pain willingness is associated with lower disability and pain interference; less depression, anxiety, stress, and isolation; and more mindfulness [28–30]. In addition, the acceptance of cancer pain is related to increased psychological well-being and decreased depressive symptoms and pain catastrophizing [31]. Therefore, paying close attention to patients' pain acceptance status and helping them to accept and acknowledge their pain could help them adapt to pain and ensure better functioning and quality of life despite chronic pain.

Although some research has been conducted on pain acceptance, few studies have examined pain acceptance and its correlates among cancer patients. The present study aimed at examining the level and correlates of pain acceptance in cancer patients from mainland China in order to understand the general pain level among cancer patients and identify patients with poor pain acceptance, which can facilitate the implementation of ACT interventions as early as possible.

## 2. Methods

### 2.1. Design

We used a cross-sectional survey design, where patients were nested in wards of Hunan Cancer Hospital, a tertiary cancer hospital in the middle south of mainland China.

### 2.2. Subjects and Procedure

Cancer patients with chronic pain consented to participate in the study in a tertiary cancer hospital in Hunan Province of China from November 2016 to May 2017. In China, hospitals are accredited as primary hospitals (community health centers), secondary hospitals (local hospitals or regional hospitals), or tertiary hospitals (comprehensive general hospitals and large specialized hospitals). Tertiary hospitals have the most highly skilled professionals and the best medical resources, which draw patients from all over China. Therefore, adequate samples were available in the hospital for our study. The inclusion criteria were that patients (1) were diagnosed with cancer; (2) were aged 18 and over; and (3) had a duration of pain >3 months, with pain everyday or almost everyday. The exclusion criteria were that patients had (1) a history of psychosis, cognitive impairment, or communication disorders or (2) participated/been involved in certain psychotherapy programs within the previous three months.

The sample size was estimated by the statistical calculation formula of a cross-sectional survey of related factors [32]. According to a previous report [21], the standard deviation of CPAQ-8 was 9.36, assuming the admissible error does not exceed 3 [33], on the basis of a two-tailed *α* at a significance level of <0.05, at least 150 subjects are required to detect for status survey and related factors analysis of CPAQ-8. The research was endorsed by the Ethics Review Committee of the Affiliated Cancer Hospital of Xiangya School of Medicine, Central South University/Hunan Cancer Hospital (No. SBQLL-2016-002). Prior to the enrolment of the study, participants were clearly informed the objectives, confidentiality considerations, and the anonymity in data collection, analysis, and report. All of the questionnaires were completed by individuals with no interference. The investigators assisted those who could not complete the questionnaires independently. The process was voluntary, and participants could choose to discontinue at any time. All participants signed an informed consent form. For the patients who scored very pathologically in both questionnaires, the psychological counselors who worked in the psychological care unit of the hospital will provide free psychological comfort and counseling.

### 2.3. Measures

All the primary data were collected by investigators of the research team. Demographic and clinical information were collected with a brief demographics survey including participants' age, gender, education, marital status, economic status, and self-perceived religiosity information. The pain information items assessed the number of pain sites, pain duration, and medication use of subjects.

The acceptance of chronic pain was measured with the 8-item Chronic Pain Acceptance Questionnaire (CPAQ-8), a validated short version of the original CPAQ 20-item scale [20]. Four items (1, 3, 5, 6) assess activity engagement (AE), and four of them (2, 4, 7, 8) evaluate pain willingness (PW). The items are rated using seven-point scale ranging from “never true” to “always true” (0 to 6). When obtaining the total score, pain willingness is reverse-scored. The maximal score of the questionnaire is 48, with higher scores indicating better acceptance of pain. The Chinese version of the CPAQ-8 has excellent reliability and validity, with an alpha coefficient 0.84, CMIN/DF = 1.832, NNFI = 0.962, CFI = 0.98, GFI = 0.967, and RMSEA = 0.061 [21].

Anxiety and depression levels were assessed with the Hospital Anxiety and Depression Scale (HADS), which is a 14-item inventory used to examine the degree of anxiety and depression disorders of patients in nonpsychiatric hospitals [34]. The HADS has two subscales—the anxiety subscale (HADS-A) and depression subscale (HADS-D)—each consisting of seven items. A four-point Likert scale (0–3) is used to rate the items. A higher score represents more severe psychological distress. This instrument is widely used in clinical settings, and the Chinese version used in the current study has sound reliability, with Cronbach's alpha coefficient 0.832. Cronbach's alpha coefficients of the HADS-A and HADS-D subscales are 0.753 and 0.764, respectively [35].

### 2.4. Data Management and Statistical Analyses

Data were entered by two persons, and the missing and outlier data were examined carefully later. Descriptive statistics were used to describe the baseline characteristics and outcome measures. We treated CPAQ-8 and HADS scores as continuous variables and baseline characteristics as nominal categorical variables. The independent sample's *t*-test and one-way analysis of variance (ANOVA) were conducted to identify patient demographic characteristics and pain information associated with pain acceptance.

To further explore the correlation between pain acceptance and HADS, we measured Pearson's product-moment correlation coefficients. All of the statistical tests were two-tailed. A *P* value <0.05 was considered statistically significant. Data analyses were performed using IBM SPSS Statistics 22 for Windows (IBM Corp., Armonk, NY).

## 3. Results

### 3.1. Sample Characteristics

**Figure 1:**
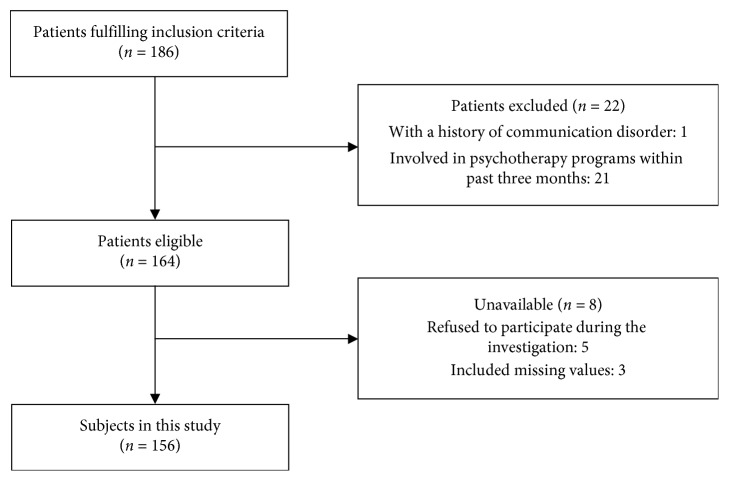
Flow chart outlining derivation of the sample.

A flow diagram outlining the selection of the study participants is shown in Figure 1. To increase the power of test, a total of 186 cancer patients were registered in the present study. One ineligible subject was excluded primarily because of having a history of communication disorder, whereas 21 ineligible subjects had participated in psychotherapy programs in the past three months. Of the eligible patients, five refused to participate during the process, and three questionnaires had missing data. Finally, a total of 156 questionnaires (response rate, 83.9%) were included in the analysis. The amount of participants could meet the estimated sample size.

Table 1 shows data on patient demographics and pain characteristics. Participants averaged 52.3 years of age (range: 24 to 77). Eighty-three (53.2%) of the patients were male, and 73 (46.8%) were female. Most of them (148, 94.9%) were married, and 150 (96.2%) were not religious. As for pain information, 134 (85.9%) of the participants had pain that lasted for 3–6 months, 135 (86.5%) had no more than three pain sites, 148 (94.9%) were using painkillers, and 140 (89.7%) had taken the analgesic for 3–6 months.

### 3.2. Relationship between Sample Characteristics and Pain Acceptance

The *t*-test (gender, marital status, self-perceived religiosity, pain duration, condition of painkillers usage, and duration of taking analgesics) and ANOVA (age, education, economic status, and number of pain sites) were used to examine the relationships between participants' general information and pain acceptance. As seen in Table 1, the results showed that victims' pain acceptance was significantly correlated with their age (*P*=0.048), gender (*P* < 0.001), marital status (*P*=0.001), pain duration (*P*=0.022), number of pain sites (*P*=0.010), and duration of taking analgesics (*P* < 0.001) but had no significant correlation with the condition of painkiller usage.

### 3.3. Correlation Analysis of Pain Acceptance and Negative Emotions

Pearson correlation coefficients were calculated to explore the relationship between pain acceptance and psychological distress. As expected, the results showed that high acceptance was associated with low anxiety and depression. The strongest relationships were found for the HADS total score and the CPAQ-8 (*r*=−0.625, *P* < 0.01), pain willingness (*r*=−0.585, *P* < 0.01), and activity engagement (*r*=−0.554, *P* < 0.01) (Table 2).

## 4. Discussion

This study aimed at exploring the level of pain acceptance among cancer patients in China, as well as its correlations with general information and HADS. We found that the average prevalence pain acceptance of Chinese cancer patients was 25.99 (8.56), which was lower than the result of 27.90 (6.55) for chronic pain participants in Australia [36] and the result of 32.78 (9.36) for noncancer chronic pain patients in China [21]. Different from other nonmalignant chronic pain, cancer pain is a complex physiological, pathological, and emotional experience. It can be related to the tumor itself, diagnostic/therapeutic procedures, or treatment-associated adverse events [37]. According to a systematic review, the overall prevalence of cancer-related pain could range from 50 to 70% [5]. In another study, 31–45% of patients rated their pain intensity as moderate or severe [38]. Cancer pain is closely linked to insomnia, fatigue, and increased perceived disability. The patients are often preoccupied by their suffering and fear of death, which would greatly impair their acceptance of pain. They find it hard to be involved in normal life, participate in value-direct activities, and pursue their own goals in the presence of pain.

Our univariate analysis showed that different sociodemographic data, including age, gender, pain duration, number of pain sites, and duration of taking analgesics, were associated with distinct levels of pain acceptance. We found that the pain acceptance of participants aged over 70 ranked the highest, followed by those aged between 51–70 and 18–30, whereas those aged 31–50 had the lowest acceptance. This finding partially differs from that of a previous study, which verified that age is positively associated with pain acceptance [39]—in other words, older patients will have better pain acceptance. One possible reason for the discrepancy is the economic and social diversity of the subjects. Some of the participants in the middle age range of 31–50 years of age were born after the one-child family planning policy of the early 1980s in China. They need to support the elderly and raise the young in their family and have undertaken tremendous burdens. Once they have been admitted to hospital as cancer patients, they will suffer from great pressure, which could influence their pain acceptance.

Compared with women, we found that men had better pain acceptance. Personal characteristics may lead to the low level of pain acceptance in female cancer patients. However, another study argued that because of close family and friends in their social life, women with CP would have greater social support, which helps to promote pain acceptance [40]. A possible explanation for the contrast is that, in the context of Chinese traditional culture, women are more likely to suffer from stigma, value vanity, and conceal their real feelings.

Furthermore, our results showed that the degree of pain acceptance decreases as the number of pain sites increases. Cancer patients who suffer from multisite pain have to make pain control their top priority and may have trouble getting on with their normal routine. The fear of pain hinders them in leading a full life. Our results also showed that participants with longer durations of pain and analgesic usage had higher acceptance scores, which is consistent with a previous study [39]. The likely reason for this is that the cancer patients gradually adapt to the change brought by pain. Patients with longer pain and analgesic usage durations have more pain control knowledge and experience; they can take active initiative to reduce the physical and psychological impact of pain in daily life and choose to spend their time in a meaningful way in their daily lives according to their own values.

Our findings suggested that the pain acceptance level of married patients is significantly lower than that of those who are single, divorced, or widowed. The reason may be that married patients have to shoulder more family responsibilities and pressures. However, most of the participants in our study (94.9%) were married. The sample size of unmarried, divorced, or widowed patients was too small to reflect the real differences in patients' levels of acceptance.

Consistent with past research [21, 41–43] and hypotheses, bivariate correlation coefficients demonstrated that AE, PW, and total pain acceptance were negatively correlated with anxiety and depression levels; that is, higher acceptance of chronic pain is closely linked with lower depression and anxiety. A survey conducted in 686 patients with chronic pain also showed that high pain acceptance can reduce anxiety and depression [44]. This might be due to pain acceptance representing the ability to engage in activities in the presence of pain without struggle [45]. In contrast, one study found that there was no significant effect of pain acceptance on depression [46]. This inconsistency may result from the heterogeneity of participants and may also be related to the small sample size of participants in that study.

Our study has several strengths. First, although many researchers have conducted surveys on chronic pain acceptance, most of the subjects had noncancer pain; furthermore, because of the differences in culture, economy, and healthcare systems, there was a need to carry out a study among secular Chinese cancer patients. To the best of our knowledge, this was the first study to explore pain acceptance among cancer patients in mainland China using CPAQ-8. Second, little has been known regarding the influencing factors of cancer-related pain acceptance, and our study helped clarify these. Third, with the proper application of our findings, early recognition and intervention for low-acceptance individuals could be developed, which would serve as an important and innovative nonpharmacological intervention for pain.

This study also has certain limitations. First, this was a cross-sectional survey conducted in a single tertiary cancer hospital. Because of the limited sample sources, whether and to what extent our subjects could represent other cancer patients in China remains to be determined. Samples from cancer and pain departments in comprehensive hospitals and community clinics should be examined in future studies. Second, the disease information, such as the tumor stage, antineoplastic treatments effect, and life prognosis, is not reported in the sample characteristics. These factors might influence the level of pain acceptance. The next steps will commit to address the potential sources of bias. Third, the recruited participants did not receive any interventions in the hospital before the survey; hence, further empirical studies should measure the pain acceptance of patients before and after interventions. Finally, cancer pain is a comprehensive experience consisting of multiple dimensions that cannot be evaluated merely by questionnaire. A qualitative design is needed to gain insight into patients' feelings, ideas, and perceptions of pain in future research.

## 5. Conclusions

The present study contributes to the understanding of Chinese cancer patients' pain acceptance. The pain acceptance of Chinese cancer patients is relatively low compared with the findings for those with nonmalignant pain. Age, gender, pain duration, number of pain sites, and duration of taking analgesics are all factors that influence pain acceptance. Depression and anxiety are negatively correlated with the acceptance of chronic pain. Based on our findings, cancer patients who are aged between 31 and 50, female, affected by multiple-site pain and a short period of pain, taking painkillers for less than three months, and in a state of anxiety or depression should receive more attention in Chinese medical settings. Based on the awareness of pain acceptance, healthcare professionals should implement effective individualized acceptance-oriented interventions such as ACT to enhance pain acceptance and alleviate negative emotions, such as depression and anxiety.

## Figures and Tables

**Table 1:** Relationships between sample characteristics and pain acceptance.

Characteristics	Total (*n*=156)		Pain acceptance (mean ± SD)	*t/F*	*P* value
	*n*	*%*			
Age (years)				2.699	0.048
18–30	9	5.8	26.4 ± 11.3		
31–50	61	39.1	23.7 ± 9.0		
51–70	75	48.1	27.4 ± 7.8		
>70	11	7.0	28.7 ± 6.6		

Gender				11.796	<0.001
Male	83	53.2	31.5 ± 5.7		
Female	73	46.8	19.7 ± 6.8		

Education				0.477	0.699
Illiteracy/primary school	28	17.9	27.5 ± 7.0		
Middle school	63	40.4	25.4 ± 8.3		
High school	52	33.3	26.2 ± 9.1		
College or above	13	8.3	25.0 ± 11.0		

Marital status				−3.517	0.001
Married	148	94.9	25.5 ± 8.3		
Unmarried	8	5.1	36.0 ± 7.3		

Religious faith				−0.002	0.999
No	150	96.2	26.0 ± 8.6		
Yes	6	3.8	26.0 ± 7.1		

Income (RMB)				0.439	0.646
<3,000	74	47.4	26.6 ± 8.7		
3,000–5,000	60	38.5	25.7 ± 8.2		
>5,000	22	14.1	24.8 ± 9.5		

Pain duration (months)				−2.320	0.022
3–6	134	85.9	25.4 ± 8.4		
>6	22	14.1	29.9 ± 8.7		

Number of pain sites				3.881	0.010
1	53	34.1	28.6 ± 7.5		
2	37	23.7	26.1 ± 8.9		
3	35	22.4	25.3 ± 8.8		
>3	31	19.8	22.3 ± 8.5		

Analgesic application				0.256	0.798
No	8	5.1	26.8 ± 10.4		
Yes	148	94.9	26.0 ± 8.5		

Duration of analgesic use (months)				6.303	<0.001
<3	9	5.8	15.3 ± 2.9		
3–6	125	80.1	26.3 ± 7.9		
>6	14	9.0	29.9 ± 10.2		

**Table 2:** Correlations between CPAQ-8 and HADS.

Anxiety and depression	Pain acceptance		
	Activity engagement	Pain willingness	CPAQ-8
HADS-A	−0.461^*∗∗*^	−0.479^*∗∗*^	−0.515^*∗∗*^
HADS-D	−0.534^*∗∗*^	−0.573^*∗∗*^	−0.608^*∗∗*^
HADS total score	−0.554^*∗∗*^	−0.585^*∗∗*^	−0.625^*∗∗*^

^*∗∗*^ *P* < 0.01 (two-tailed). CPAQ-8: 8-item Chronic Pain Acceptance Questionnaire; HADS: Hospital Anxiety and Depression Scale.

## Data Availability

The data used to support the findings of this study are available from the corresponding author upon request.
